# Niche dynamics of *Memecylon* in Sri Lanka: Distribution patterns, climate change effects, and conservation priorities

**DOI:** 10.1002/ece3.8415

**Published:** 2021-12-03

**Authors:** Prabha Amarasinghe, Narayani Barve, Hashendra Kathriarachchi, Bette Loiselle, Nico Cellinese

**Affiliations:** ^1^ Department of Biology University of Florida Gainesville Florida USA; ^2^ Florida Museum of Natural History University of Florida Gainesville Florida USA; ^3^ Biodiversity Institute University of Florida Gainesville Florida USA; ^4^ Cooperative Agricultural Research Center Prairie View A&M University Prairie View Texas USA; ^5^ Department of Plant Sciences University of Colombo Colombo Sri Lanka; ^6^ Department of Wildlife Ecology and Conservation University of Florida Gainesville Florida USA; ^7^ Tropical Conservation and Development Program Center for Latin American Studies Gainesville Florida USA

**Keywords:** climate change, ecological niche models, gap analysis, habitat suitability, *Memecylon*, Sri Lanka

## Abstract

Recent climate projections have shown that the distribution of organisms in island biotas is highly affected by climate change. Here, we present the result of the analysis of niche dynamics of a plant group, *Memecylon*, in Sri Lanka, an island, using species occurrences and climate data. We aim to determine which climate variables explain current distribution, model how climate change impacts the availability of suitable habitat for *Memecylon*, and determine conservation priority areas for Sri Lankan *Memecylon*. We used georeferenced occurrence data of Sri Lankan *Memecylon* to develop ecological niche models and assess both current and future potential distributions under six climate change scenarios in 2041–2060 and 2061–2080. We also overlaid land cover and protected area maps and performed a gap analysis to understand the impacts of land‐cover changes on *Memecylon* distributions and propose new areas for conservation. Differences among suitable habitats of *Memecylon* were found to be related to patterns of endemism. Under varying future climate scenarios, endemic groups were predicted to experience habitat shifts, gains, or losses. The narrow endemic *Memecylon* restricted to the montane zone were predicted to be the most impacted by climate change. Projections also indicated that changes in species’ habitats can be expected as early as 2041–2060. Gap analysis showed that while narrow endemic categories are considerably protected as demonstrated by their overlap with protected areas, more conservation efforts in Sri Lankan forests containing wide endemic and nonendemic *Memecylon* are needed. This research helped clarify general patterns of responses of Sri Lankan *Memecylon* to global climate change. Data from this study are useful for designing measures aimed at filling the gaps in forest conservation on this island.

## Graphical abstract

With the aim of understanding how climate change affects the distribution of island‐dwelling plants, this research used occurrence data of a Sri Lankan woody plant group, *Memecylon*, to develop ecological niche models and assess future potential distributions under six climate change scenarios. Our results provided insights into patterns of responses of Sri Lankan *Memecylon* to global climate change. Additionally, data from this study are useful for designing measures aimed at filling the gaps in forest conservation on this island.

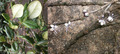

## 1 INTRODUCTION

Island ecosystems have received much attention in climate change research because they are considered to be among the most vulnerable (Harter et al., 2015; Leclerc et al., 2020; Taylor & Kumar, 2016). Such island vulnerability can be results of extreme weather events that lead to the displacement of suitable habitats of species and the island conditions (e.g., size and restriction from the surrounding ocean) that limit organisms' response to climate change (Harter et al., 2015; Veron et al., 2019). Therefore, understanding how climate change and human‐induced habitat loss will influence the risk of species extinction is critical for informing environmental policies (Cooper et al., 2011; Sinervo et al., 2010) on these islands.

**FIGURE 1:**
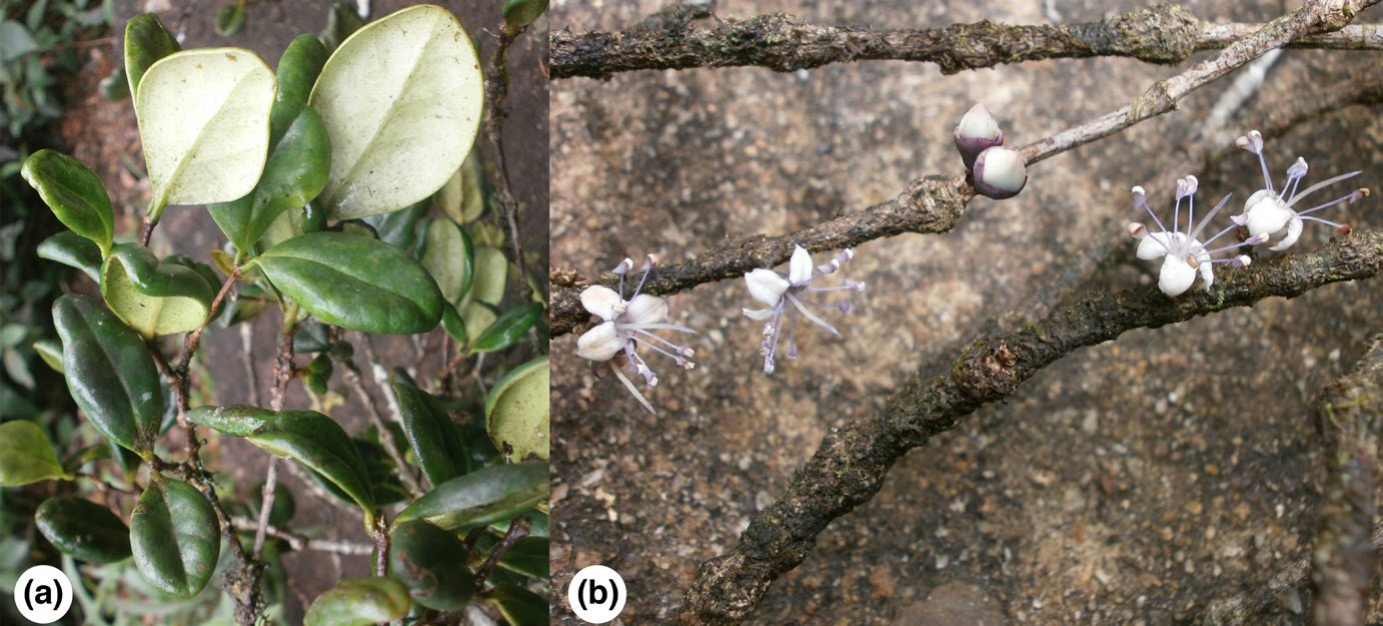
*Memecylon rotundatum* in Sri Pada Mountain, Central Province, Sri Lanka. (a) vegetative and (b) reproductive morphology. Photography: Prabha Amarasinghe

Occurrence data on species distribution and high‐resolution spatial data on climate can be integrated to predict climatic dimensions of a species niche, which is known as Ecological Niche Modeling (ENM) (Hijmans et al., 2005; Peterson, 2011). These models use a growing number of quantitative approaches to approximate fundamental niches of species in relation to temperature, precipitation, and other associated climate and topographic variables (Elith & Leathwick, 2009; Randin et al., 2009), and these ENMs can be used to develop conservation strategies (Andrade‐Díaz et al., 2019; Carroll, 2010). Using these tools, we examined ENMs of a woody plant group, *Memecylon* L. in Melastomataceae (Figure 1), on a continental island, Sri Lanka, and predicted its response to future climate scenarios in order to inform conservation priorities.

### 1.1 Climate, vegetation, and land use of Sri Lanka

**FIGURE 2:**
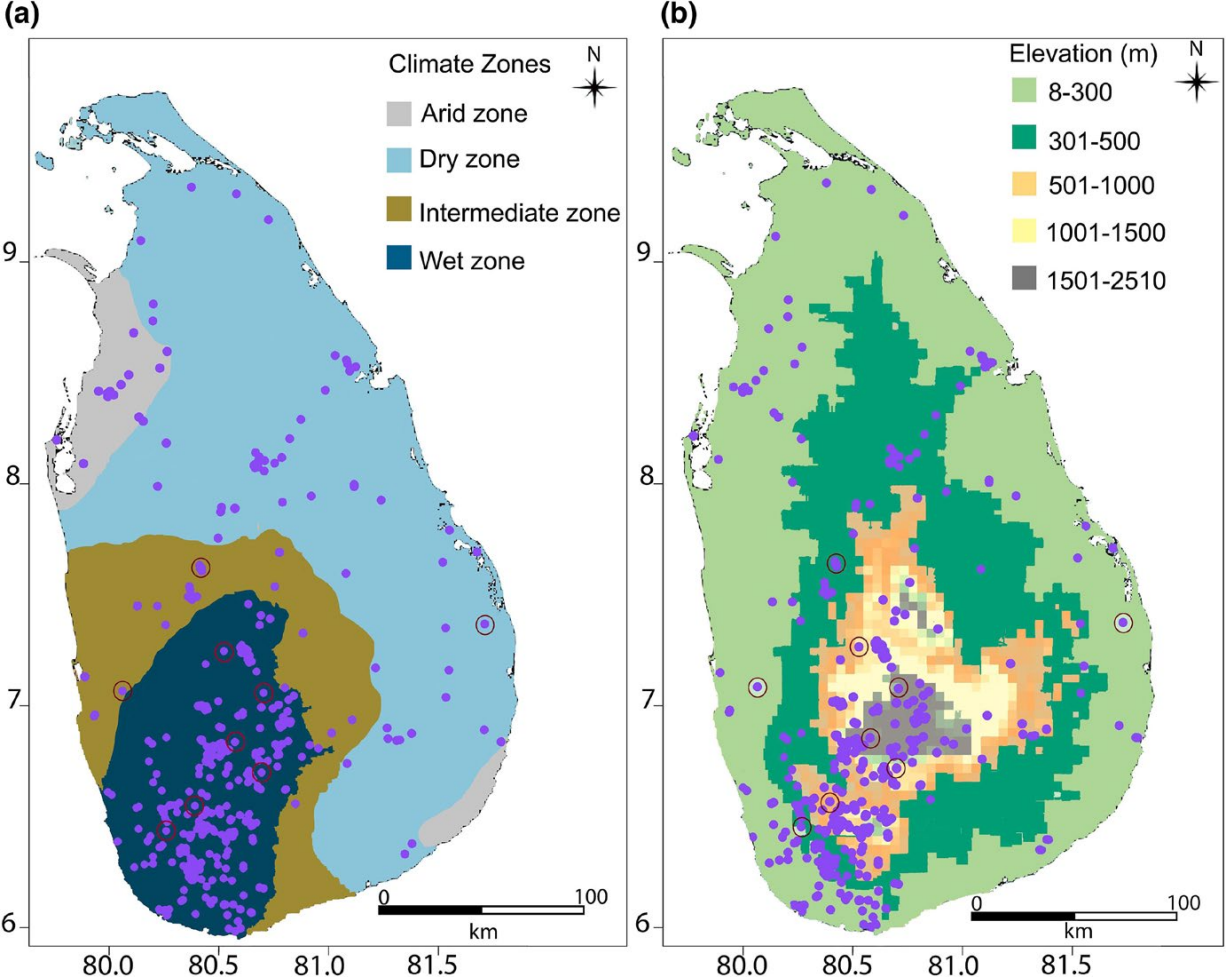
(a) Climate zones (b) elevation zones. Purple dots show *Memecylon* occurrences. Maroon circles show the field collection sites

The climate of Sri Lanka is dominated by two monsoon seasons known as southwest and northeast monsoons (Bonnefille et al., 1999). Based on the distribution of mean annual rainfall, three major precipitation zones have been recognized (Figure 2): (1) wet zone: >2500 mm of rainfall; (2) dry zone: <1750 mm; and (3) intermediate zone: 2500–1750 mm (Ashton et al., 1997). Additionally, two small areas at the extreme northwest and southeast of the island have arid climates. Sri Lanka's near‐equator position provides a tropical climate with a mean annual temperature ranging from 15°C in high to 28°C in low elevations (Silva & Sonnadara, 2016).

The varied climates in Sri Lanka have resulted in different ecosystems that harbor a wide range of floristic diversity (Ashton et al., 1997). For example, the wet zone contains tropical rainforests, with broad‐leaved trees and lianas; the intermediate zone contains broad‐leaf forest with an undergrowth of shrubs; dry zone forests consist of shrubs with deciduous leaves; and the arid zone contains open scrubland with scattered trees (Erdelen, 1988). A significant feature of plant diversity in Sri Lanka is the remarkably endemic angiosperm flora with approximately 863 endemic species out of circa 3087 (National Red List, 2020). Although the close biotic affinities between Sri Lanka and India have been identified (Pethiyagoda & Sudasinghe, 2017), there is also evidence for a distinct biotic component uniquely assembled on the island (Bossuyt, 2004).

Similar to other tropical forests around the world, those in Sri Lanka are threatened by the impacts of land‐use changes (Dissanayake, 2020; Dissanayake et al., 2019). Yet, the ecological consequences of these anthropogenic changes are understudied relative to other forests of the New World and Old World Tropics (Gopal, 2013). Approximately, one‐third of the total land area in Sri Lanka is used for agriculture, and locations that are in close proximity to district capitals are impacted due to increasing population pressure and urbanization. The reduction of forest cover has accelerated due to infrastructure enhancement, economic reforms, and population redistribution (Rathnayake et al., 2020). However, limited data on vegetation cover to compare with recent land‐use information impede effective environmental management and planning on this island.

### 1.2 *Memecylon* in Sri Lanka

There are 32 *Memecylon* species in Sri Lanka, and of them, 25 are reported as endemic (Bremer, 1988). Sri Lankan *Memecylon* is distributed in the arid, dry, wet, and intermediate zones, in a wide range of habitats (Figure 2) (Global Biodiversity Information Facility, GBIF: http://data.gbif.org/species/); Bremer, 1988). Some *Memecylon* species are endemic and rare (e.g., *Memecylon revolutum* Thwaites), while a few others, such as *Memecylon umbellatum* Burman, *Memecylon capitellatum* Linnaeus, and *Memecylon sylvaticum* Thwaites, have island‐wide distribution in dry, intermediate, and wet climatic areas. The remaining *Memecylon* are confined to wet or dry forests. *Memecylon* also shows a wide range of ecological diversity in different locations, including the highest mountain of the island, Pidurutalagala (e.g., *Memecylon cuneatum* Thwaites), the dry lowland forests (e.g., *M. umbellatum*), and coastal areas (e.g., *M. capitellatum*). Due to its diversity across the island and high regional endemism, this plant group represents an ideal model to investigate niche differentiation and the possible impact of climate change on vegetation in different climate zones. Currently, Sri Lanka has set aside a considerable proportion of land for conservation (UNEP‐WCMC & IUCN, 2020). However, it is important to understand if these conserved areas capture diverse habitats of rare and endemic species. *Memecylon*, which has a relatively large number of species characterized by high endemism, is suitable as a model system to investigate whether the current conservation measures adequately capture the diversity of areas and estimate the potential loss of species' suitable areas under varying climate change scenarios.

To date, research on *Memecylon* has mainly focused on its taxonomy, ethnobotany, and evolution (e.g., Amarasinghe, Joshi, et al., 2021; Bremer, 1988; Sivu et al., 2013) and studies to examine how climate change potentially impacts its distribution are completely lacking. In this study, we compiled occurrences of *Memecylon* from diverse sources and bioclimatic data associated with various climate scenarios to address three key questions: (1) What are the patterns of distribution of Sri Lankan *Memecylon* under current climate conditions? (2) How will climate change impact the availability of suitable habitats of *Memecylon*, and what will be the main driving factors of these changes? And (3) Which areas should be targeted for conservation as indicated by *Memecylon* distribution in the face of climate change in Sri Lanka? Endemic species are reported as highly vulnerable to climate change (Loarie et al., 2008; Manes et al., 2021; Thuiller et al., 2006) due to occupying specialized niches, limited dispersal capabilities, and reduced adaptive capacities when compared to nonendemic species (Chichorro et al., 2019; Staude et al., 2020). Further, organisms that have more restricted geographical ranges are at greater risk (Elsen & Tingley, 2015; Stubbs et al., 2018) compared to species with large geographical ranges, which may find refugia in parts of their range (Lucas et al., 2019). Therefore, we hypothesized that endemic *Memecylon* with restricted distribution ranges in Sri Lanka may be at more risk with climate change compared to nonendemics and species with large geographic ranges. Here, we tested the null hypothesis that endemic and nonendemic *Memecylon* in Sri Lanka with all types of geographical ranges are equally vulnerable.

## 2 METHODS

### 2.1 Occurrence data collection

The study area, Sri Lanka, is located between 5°55′–9°51′N latitude and 79°52′–81°51′E longitude (WGS 84‐UTM Zone 44N) in the Indian Ocean and has a land extent of 65,525 km^2^ (Rathnayake et al., 2020). It contains three elevation zones (Figure 2) known as (1) lowland: up to 300 m above sea level; (2) upland: 300–1000 m; and (3) highland: >1000 m (Katupotha, 2013) where forests within them are broadly categorized as lowland and montane forests (Werner & Balasubramanium, 1992). Occurrence records of *Memecylon* from Sri Lanka were collected from the following herbaria: B, BM, BR, FLAS, K, L, M, MO, NY, PDA, SING, and US (acronyms: Thiers, 2020), GBIF, and published literature (Ekanayake et al., 2014; Gunathilaka, 2019; Madurapperuma et al., 2014; Medawatte et al., 2011). Upon review, identifications of some herbarium specimens were found to be erroneous: ~15% of specimens in GBIF, ~13% deposited at the PDA, ~2% at the SING, ~5% at the US Herbaria. The likely reason for the misidentification of *Memecylon* specimens is due to many specimens being sterile because of seasonal and/or rarity of flowering events (Amarasinghe, Joshi, et al., 2021). Therefore, we corrected all misidentified specimens stored in the PDA, SING, and US herbaria by carefully studying the type specimens and taxonomic descriptions during the visits to these herbaria. Other herbaria stored mostly duplicates of specimens deposited at PDA; however, when nonduplicate specimens were found from the online databases of other herbaria, occurrence data were used only from correctly identified specimens. We also selected GBIF data points that were correctly identified, based on digitized specimens.

Additional GPS points from plant locations were collected during fieldwork from June to August 2017 from randomly selected forests representing several climate and elevation zones of Sri Lanka (Figure 2: dry lowland—Ampara; intermediate lowland—Doluwakanda; wet lowland—Kanneliya, Pilikuththuwa, Sinharaja; wet montane—Peak Wilderness, Hanthana, Pidurutalagala, Sri Pada). Twenty‐five specimens from fieldwork were deposited at PDA.

In total, 903 digitized herbarium specimens were georeferenced using GeoLocate, and locations were verified by cross‐checking with Acme Mapper v2.2. (1991). All identical points (duplicate specimens which could not be detected manually), points without environmental data, and proximate data points (points that fall in the same raster cell, ~1 km^2^) were removed using R packages *spocc*, *scrubr*, and *spatstat* (Baddeley et al., 2015; Chamberlain, 2020; Chamberlain et al., 2021) on R v3.3.1 (R Core Team, 2019). Finally, based on our knowledge of *Memecylon*, all occurrence data were visually examined in QGIS v3.3.3k to look for potential errors. Samples collected from sites that are likely to be visited more frequently (i.e., near roads, urban areas, and botanic gardens) may introduce bias because those occurrence points may not adequately capture the range of environmental conditions in which a species might occur (Rocchini & Garzon‐Lopez, 2017). We believe this bias is minimal in this study because specimens and field‐collected data were primarily from locations within old‐growth or secondary forests rather than readily accessible areas. *Memecylon angustifolium* Wight, *Memecylon ellipticum* Thwaites, *Memecylon giganteum* Alston, *Memecylon leucanthemum* Thwaites, *Memecylon macrophyllum* Thwaites, *M. revolutum*, and *Memecylon wightii* Thwaites, were removed due to insufficient sampling for model generation (fewer than 10 occurrences or prone to overfitting). We also removed two species (*Memecylon gracilimum* Alston and *Memecylon macrocarpum* Thwaites) in which occurrence data were found only from a single location. Using these filtering criteria, 21 *Memecylon* taxa were used for analyses (Table 1); these selected taxa occupy different habitats, climatic zones, and elevations in Sri Lanka.

**TABLE 1:** Distribution and endemism categories of 23 *Memecylon* in Sri Lanka, and the number of occurrences (after data cleaning) used to generate niche models for each species

Species	Distribution	Category	Occurrence points
*M. capitellatum* Linnaeus	Dry zone lowlands, coastal	Nonendemic dry zone	25
*M. clarkeanum* Cogniaux	Wet zone lowlands	Nonendemic wet zone	47
*M. cuneatum* Thwaites	Montane	Narrow endemic‐montane	15
*M. discolor* Cogniaux	Wet zone lowlands	Narrow endemic‐lowland	18
*M. fuscescens* Gamble	Wet zone lowlands	Narrow endemic‐lowland	17
*M. grande* Retzius	Wet zone lowlands	Nonendemic wet zone	25
*M. hookeri* Thwaites	Wet zone lowlands	Nonendemic wet zone	18
*M. orbiculare* Thwaites	Wet zone lowlands	Narrow endemic‐lowland	20
*M. ovoideum* Thwaites	Montane	Narrow endemic‐montane	17
*M. parvifolium* Thwaites	Montane	Narrow endemic‐montane	16
*M. petiolatum* Trimen ex Alston	Dry zone lowlands	Nonendemic dry zone	21
*M. procerum* Thwaites	Wet zone lowlands	Narrow endemic‐lowland	18
*M. rhinophyllum* Thwaites	Wet and intermediate zones	Wide endemic	21
*M. rivulare* Bremer	Wet zone lowlands	Narrow endemic‐lowland	35
*M. rostratum* Thwaites	Wet zone lowlands	Narrow endemic‐lowland	25
*M. rotundatum* Thwaites (Cogniaux)	Wet zone lowland and montane	Narrow endemic‐montane	15
*M. royenii* Blume	Wet zone low‐medium elevations	Wide endemic	20
*M. sylvaticum* Thwaites	Dry zone and wet zone	Wide endemic	32
*M. umbellatum* Burman	Dry and arid zones low elevations	Nonendemic dry zone	30
*M. urceolatum* Cogniaux	Dry to intermediate zone	Wide endemic	20
*M. varians* Thwaites	Wet zone lowlands	Narrow endemic‐lowland	31

### 2.2 Climate data

All bioclimatic variables (Table 2) for the current climate at 30 arc‐second resolution were downloaded from the WorldClim 2 Global Climate Data website v.2.1 (Fick & Hijmans, 2017). We established a buffer zone of 100 km around the occurrence data of each species separately using QGIS to generate calibration areas for the models (Barve et al., 2011). We then developed spatial analyses for each species using extents that included occurrence distributions and buffer area, as well as extents based on the entire island; these analyses were done with R packages *maptools* and *mapproj* (Bivand & Lewin‐Koh, 2020; Brownrigg & Minka, 2020). Pairwise Pearson's correlation coefficients (*r*) for all bioclimatic variables within the species‐specific buffer zones were estimated to avoid collinearity between them using R packages *raster* and *rgdal* (Hijmans, Etten, et al., 2021; Keitt et al., 2010); species‐specific predictors were retained based on a threshold of |*r*| ≤ 0.65.

**TABLE 2:** Bioclimatic variables (Hijmans et al., 2005) indicated as used or not used (based on collinearity analysis) for model generation

BioClim Code	Variable name and description	Used or not
bio1	Annual mean temperature	Not
bio2	Mean diurnal range (mean of monthly (maximum temp − minimum temp))	Used
bio3	Isothermality (bio2/bio7) (×100)	Used
bio4	Temperature seasonality (standard deviation × 100)	Used
bio5	Maximum temperature of warmest month	Used
bio6	Minimum temperature of coldest month	Not
bio7	Temperature annual range (bio5–bio6)	Used
bio8	Mean temperature of wettest quarter	Used
bio9	Mean temperature of driest quarter	Not
bio10	Mean temperature of warmest quarter	Not
bio11	Mean temperature of coldest quarter	Used
bio12	Annual precipitation	Not
bio13	Precipitation of wettest month	Used
bio14	Precipitation of driest month	Not
bio15	Precipitation seasonality	Not
bio16	Precipitation of wettest quarter	Not
bio17	Precipitation of driest quarter	Not
bio18	Precipitation of warmest quarter	Used
bio19	Precipitation of coldest quarter	Used

To model future climate scenarios, we used bioclimatic variables (the year 2050—average for 2041–2060 and year 2070—average for 2061–2080) from General Circulation Models (GCMs): Beijing Climate Center Climate System Model (BCC‐CSM1‐1) and Model for Interdisciplinary Research on Climate (MIROC5) and for each, we used 2.6, 4.5, and 8.5 Representative Concentration Pathways (RCPs) (30 arc‐second resolution); these data were obtained from WorldClim v.1.4 (Hijmans et al., 2005). The GCMs, MIROC5, and BCC‐CSM1‐1 were used in this study because these GCMs capture various features of future climate for the South Asian region (Pramanik et al., 2018; Sharmila et al., 2015). Climate scenarios used were selected to include both optimistic and pessimistic events. For example, RCP 2.6 represents an optimistic future scenario and assumes an increased global mean temperature of <2°C, cumulative emissions of greenhouse gases that will peak in 2050 and then decline moderately, and eventually will be reduced by 70% by 2100 (van Vuuren, Stehfest, et al., 2011). RCP 4.5 represents an intermediate future scenario which assumes CO_2_ concentration of 650 ppm and average temperature increase of 1.0–2.6°C by the year 2100 and rapid economic growth combined with the reduction of emissions (Puchałka et al., 2021; Roeckner et al., 2011). In contrast, RCP 8.5 represents a more pessimistic scenario that assumes a CO_2_ concentration of 1350 ppm and an average temperature increase of 2.6–4.8°C during the same time frame as other models and rapid economic growth combined with intensive use of fossil fuel (Knutti & Sedláček, 2013; Puchałka et al., 2021; van Vuuren, Edmonds, et al., 2011).

### 2.3 Ecological niche models

We used MaxEnt v3.4.1 (Phillips et al., 2017) to construct Ecological Niche Models (ENMs) for each Sri Lankan *Memecylon* species because this method is designed to work with presence‐only data and has been found to perform well with sample sizes as low as 10, a useful feature when studying rare *Memecylon* (Hernandez et al., 2006; Pearson et al., 2007). To prevent model overfitting, instead of using MaxEnt default settings, we implemented a MaxEnt tuning process, which uses different combinations of model settings with the R package *ENMeval* (https://github.com/jamiemkass/ENMeval; Muscarella et al., 2014). To select the modeling parameters which give the best trade‐off between model performance and complexity, MaxEnt was tuned with all possible combinations of different feature classes (linear, quadratic, and hinge) and regularization multipliers ranging from 1 to 5, with 0.5 intervals for each species. The parameters that scored the lowest AICc values were selected (Muscarella et al., 2014). The final model was created with the best‐selected parameter set using 20 replicates, logistic output format (fits likelihood across the landscape), bootstrap resampling (for validation of all models), and 5000 maximum iterations. Here, occurrence points were subset by MaxEnt for model testing into training (70%) and testing (30%) data. The bootstrap setting was used within the training data set for all species. Because our data sets contained only presence data, 10,000 background points were randomly chosen from the study area. A jackknife test was implemented to measure the bioclimatic variable contributions to the output models (following Shcheglovitova & Anderson, 2013).

Future distributions of *Memecylon* were projected across the island using data from eight climate‐driven models run with three future greenhouse gas concentration scenarios (RCP 2.6, RCP 4.5, and RCP 8.5) applied to two GCMs (BCC‐CSM1‐1 and MIROC5) over two time periods (2050 and 2070) (selection of these models were explained under climate data selection). Projections were carried out on MaxEnt extrapolating into parts of environmental space from which there are no training samples (Owens et al., 2013; Peterson, 2011; Zurell et al., 2012). ENM algorithms have various extrapolation strategies which are broadly classified into truncation, clamping, and actual extrapolation (Owens et al., 2013). Out of them, we selected the clamping option on MaxEnt. This option uses the marginal values in the calibration area as a prediction for more extreme conditions in transfer areas (Qiao et al., 2019).

### 2.4 Statistical analysis

Ecological niche models were used to calculate the niche breadth (Connor et al., 2018). The measure of niche breadth derived by Levin's index shows the breadth of suitable climatic factors for a species at a 0–1 scale (Feinsinger et al., 1981). Here, values closer to 1 reflect generalist species with wide climatic tolerance, while values closer to 0 represent more specialized species (Feinsinger et al., 1981).

The models were converted into binary presence–absence maps with three threshold approaches: the minimal training presence threshold, the threshold that equalizes sensitivity and specificity, and the threshold that maximizes the sum of sensitivity and specificity of the binary maps using R packages *scales* (Wickham & Seidel, 2020). Cohen's KAPPA values were calculated for threshold models using the R package *ecospat* (Broennimann et al., 2020) to evaluate the model performance. KAPPA is a threshold‐dependent matrix of model evaluation and ranges from −1 to +1. Generally, the values of KAPPA below 0.4 indicate poor model performance, while values above 0.4 indicate good to excellent model performance (Ahmad et al., 2019). Based on KAPPA results, we selected the “best” models for downstream analyses. These threshold models (i.e., binary maps) were used to calculate habitat suitability for *Memecylon* under the current environmental conditions compared with projections based on future scenarios to examine changes in habitat suitability.

The Multivariate Environmental Similarity Surface (MESS) (Elith et al., 2010) for each species was performed to compare the current and future climates at each locality and the MESS index was estimated using the R package *dismo* (Hijmans, Phillips, et al., 2021).

### 2.5 Patterns in endemic categories

Sri Lankan *Memecylon* were classified into five categories based on endemism information (Bremer, 1988; Sivu et al., 2012; Subramanyam & Rao, 1949; The National Red List, 2020), area of suitable habitats, elevation, climate variables of the current distribution, and niche breadth; these categories were wide endemic, lowland narrow endemic, montane narrow endemic, dry zone nonendemic, and wet zone nonendemic. Endemic *Memecylon* that have >10,000 km^2^ area of suitable habitats and >0.5 niche breadth were categorized as wide endemics. *Memecylon* were classified as being narrow endemics if their current geographic projection of ecological niche was <10,000 km^2^ and niche breadth was <0.5. This narrow endemic category was subclassified as montane narrow endemics (occurrence points are restricted to >300 m above sea level) and lowland narrow endemic (occurrence points are distributed in lowland, i.e., <300 m above sea level). Nonendemic *Memecylon* which contain suitable habitats within the wet zone of the island and showed a strong contribution of precipitation‐related bioclimatic variables were subclassified as wet zone nonendemics. Nonendemic *Memecylon* which contain suitable habitats within the dry zone and showed a strong contribution of temperature‐related bioclimatic variables were subclassified as dry zone nonendemic. By the above‐defined standards, we found four species to be wide endemics, eight as lowland narrow endemics, three as montane narrow endemics, and an additional three species each were categorized as wet‐zone and dry‐zone nonendemics, respectively.

In each endemic category, the threshold models of species were overlapped on each other and the overlapping area under the current condition was calculated using customized R scripts. These overlaps showed regions where niche conditions are suitable for two or more taxa. Threshold models of each taxon in 12 future models were also overlapped using the above procedure, and changes in the distribution of suitable habitat (km^2^) for each species between current and future distributions were evaluated.

### 2.6 Gap analysis

A gap analysis (Scott et al., 1993) was conducted to evaluate conservation priorities based on existing protected areas and *Memecylon* occurrences. First, to identify the geographic patterns of *Memecylon* species richness (area showing the maximum overlap of suitable habitat), we overlapped current individual suitable areas of *Memecylon* species by endemic categories. The resulting raster files of species richness were reclassified to indicate the number of species in each grid cell using the R package *raster* (Hijmans, Etten, et al., 2021) in customized R scripts. Then, a recently developed high‐resolution land‐cover map of Sri Lanka (Rathnayake et al., 2020) was used to extract forest layers with QGIS. The shapefiles of forest covers were converted to the same projection as species richness GIS layers. The reprojected forest cover map was cropped to the extent of the species richness layer. Next, the area of the cropped forest cover was calculated with QGIS. From that, we examined how well forest cover is represented in the richness maps of *Memecylon* in each endemic category.

A protected areas map of Sri Lanka from the World Database on Protected Areas (WDPA) (UNEP‐WCMC & IUCN, 2020) was used as a baseline to assess to what extent *Memecylon* were captured by current conservation efforts. Two conservation categories of the protected areas map, (1) conservation forest category and (2) all conserved (this includes conservation forests, Ramsar sites, and UNESCO man and the biosphere reserves, world heritage sites, and strict natural reserves) category, were extracted and separately merged using QGIS. The resulting conservation maps were cropped using QGIS to fit the extent of the richness map. Percent areas of conservation forests and all conserved areas were then estimated using the raster calculator in QGIS. By comparing the forest cover and protected area results, the area of unprotected forest falling within the richness area in each *Memecylon* category was estimated. This comparison was repeated for the predicted future (2050 and 2070) richness areas based on the most optimistic climate scenarios using current protected area maps; this analysis assumes that the current protected areas remain the same for 2050 and 2070. Finally, the unprotected forests which require conservation were determined by superimposing the current richness maps of all endemism categories of *Memecylon*, forests cover, and protected area maps on QGIS.

## 3 RESULTS

### 3.1 Occurrence data

Of 928 occurrence records compiled, 486 occurrence points were recovered after cleaning and selecting 21 species for analysis. The number of cleaned occurrence points per species ranged from 15 to 47 for the selected *Memecylon* taxa. The species were mostly distributed in the wet zone, in the southwest area of the island, but five were distributed in the dry zone, and two were from the intermediate zone of Sri Lanka. Average elevation ranged from 30 m (e.g., *M. capitellatum*) to 2524 m (e.g., *M. cuneatum*) (Figure 2 and Table 1).

### 3.2 Current suitable habitats

When niche space is projected into geographic space, *Memecylon* species tend to occupy distinct suitable habitats under current conditions. These ENMs, which approximate predicted fundamental niches of each species, showed different contributions of bioclimatic variables as indicated by area under the receiver operating characteristic curve (AUC: with and without the contributing variables) and permutation importance (Table 3). Mean temperature of the coldest quarter (bio11) is the variable, which contributed to models of most species (17 out of 21).

**TABLE 3:** Bioclimatic variable contribution based on jackknifing (AUC with and without contributing variables) and permutation importance

Species	FC	RM	Selected bioclimatic variables	AUC without the variable	AUC only with the contributing variable	Permutation importance (%)
*M. capitellatum*	LQ	2.5	bio2	0.67	0.64	20.6
			bio4	0.63	0.67	77.1
			bio11	0.70	0.39	2.1
*M. clarkeanum*	LQH	4	bio3	0.92	0.79	10.6
			bio4	0.91	0.85	50.8
			bio 7	0.91	0.71	23.5
			bio11	0.89	0.70	12.4
			bio13	0.93	0.80	2.5
*M. cuneatum*	L	1	bio3	0.99	0.93	6.8
			bio 7	0.99	0.73	0.1
			bio11	0.99	0.98	13.0
			bio19	0.99	0.95	79.9
*M. discolor*	LQ	2.5	bio3	0.93	0.75	1.4
			bio 4	0.92	0.79	32.1
			bio 7	0.92	0.75	9.6
			bio11	0.86	0.85	56.6
*M. fuscescens*	L	1	bio5	0.89	0.70	9.4
			bio7	0.84	0.82	63.7
			bio11	0.84	0.76	5.2
			bio18	0.85	0.84	26.8
*M. grande*	L	1	bio3	0.93	0.52	3.1
			bio7	0.90	0.79	38.9
			bio8	0.92	0.50	1.3
			bio18	0.88	0.90	56.5
*M. hookeri*	L	1	bio3	0.87	0.64	1.1
			bio4	0.83	0.88	64.7
			bio7	0.89	0.82	34.1
*M. orbiculare*	LQ	1	bio4	0.93	0.86	63.5
			bio7	0.94	0.94	4.12
			bio11	0.90	0.77	19.9
			bio19	0.93	0.81	12.39
*M. ovoideum*	L	1	bio3	0.98	0.88	1.5
			bio7	0.98	0.78	0.1
			bio11	0.91	0.98	92.0
			bio19	0.98	0.88	6.2
*M. parvifolium*	L	1	bio3	0.97	0.84	12.1
			bio7	0.97	0.77	0.04
			bio11	0.82	0.97	87.3
			bio19	0.92	0.61	0.39
*M. petiolatum*	L	2.5	bio2	0.74	0.62	19.3
			bio13	0.62	0.74	80.6
*M. procerum*	L	1	bio3	0.89	0.67	8.3
			bio4	0.90	0.88	45.9
			bio7	0.90	0.84	23.3
			bio19	0.89	0.80	22.4
*M. rhinophyllum*	L	1	bio3	0.82	0.59	1.5
			bio4	0.63	0.82	97.1
			bio11	0.82	0.60	1.3
*M. rivulare*	LQH	4	bio3	0.94	0.77	1.7
			bio4	0.93	0.89	15.9
			bio7	0.93	0.81	10.5
			bio11	0.91	0.68	10.5
			bio13	0.92	0.89	61.1
*M. rostratum*	LQH	2.5	bio3	0.88	0.75	13.4
			bio4	0.76	0.87	80.9
			bio11	0.87	0.52	5.5
*M. rotundatum*	L	1	bio3	0.95	0.69	0.043
			bio4	0.93	0.77	74.45
			bio11	0.90	0.95	22.8
			bio19	0.96	0.81	2.64
*M. royenii*	L	2.5	bio3	0.81	0.63	20.7
			bio4	0.64	0.81	71.5
			bio11	0.82	0.52	7.6
*M. sylvaticum*	L	1	bio4	0.69	0.76	76.2
			bio11	0.76	0.65	5.0
			bio13	0.78	0.66	18.6
*M. umbellatum*	LQH	1	bio3	0.67	0.62	14.3
			bio4	0.62	0.67	80.0
			bio11	0.50	0.50	5.6
*M. urceolatum*	L	1	bio4	0.75	0.76	68.07
			bio11	0.78	0.73	20.63
			bio19	0.84	0.57	11.29
*M. varians*	LQH	2.5	bio3	0.89	0.79	11.4
			bio7	0.81	0.76	51.18
			bio11	0.84	0.66	25.08
			bio13	0.91	0.71	12.2

Note

FC, Feature Class (L: linear, Q: quadratic, H: hinge); RM, Regularization Multiplier.

Selected bioclimatic variables are species‐specific predictors.

AUC scores of the resultant models are not reported in this study as they should be interpreted with caution because sampling bias can result in spatial clustering of points, which may affect model quality by inflating model accuracy (Veloz, 2009). Therefore, we assessed model performance using Cohen's KAPPA statistics, since much of our analyses and comparisons were based on binary maps (i.e., threshold selected to convert mat to areas of suitable and not suitable habitat) and a threshold‐dependent measure, like KAPPA, is more suitable for these maps. Of the three threshold approaches examined, we selected the threshold approach that equalized sensitivity and specificity because of higher overall model performance (see also Bean et al. (2012) and Shabani et al. (2018)). Performance scores of threshold ENMs (Table 4) under the current distribution showed the majority had good performance (KAPPA > 0.4); a few, however, showed poor performance (KAPPA < 0.4) (Ahmad et al., 2019). We caution interpretation of model performance results given that presence localities used for test points may be clustered in space and, therefore, not totally independent (Roberts et al., 2017). Further, due to scarce data (further subsetting of occurrence data of some species was not possible) and some species occur only over small spatial scales, we could not employ techniques that use spatial block cross‐validation (Roberts et al., 2017). These shortcomings may inflate KAPPA performance measures.

**TABLE 4:** Extent (km^2^) of suitable habitat for each species after assigning threshold values under current and future climate conditions

Taxon	Current suitable habitats	Niche breadth	Future suitable habitats											
			2050						2070					
			B2.6	B4.5	B8.5	M2.6	M4.5	M8.5	B2.6	B4.5	B8.5	M2.6	M4.5	M8.5
*M. capitellatum*	20,169 [0.42]	0.81	33,562 (64)	28,055 (39)	34,954 (73)	2447 (21)	25,455 (26)	25,206 (24)	27,147 (34)	24,254 (20)	26,568 (31)	33,561 (66)	29,072 (44)	26,519 (31)
*M. clarkeanum*	5656 [0.56]	0.23	22,846 (30)	7505 (32)	0 (−100)	16,923 (199)	15,392 (172)	8805 (55)	20,990 (271)	15,280 (170)	7013 (23)	21,566 (281)	18,252 (222)	8799 (55)
*M. cuneatum*	4812 [0.11]	0.11	0 (−100)	0 (−100)	0 (−100)	0 (−100)	0 (−100)	0 (−100)	0 (−100)	0 (−100)	0 (−100)	0 (−100)	0 (−100)	0 (−100)
*M. discolor*	6094 [0.49]	0.11	10,393 (70)	19,087 (213)	19,087 (213)	24,509 (302)	11,845 (94)	8510 (39)	20,172 (231)	19,966 (227)	9572 (57)	15,093 (147)	12,986 (113)	11,565 (89)
*M. fuscescens*	5852 [0.29]	0.49	3355 (−43)	5862 (0)	5657 (−3)	161 (−97)	3881 (−33)	2952 (−49)	1427 (−76)	5146 (−12)	2360 (−60)	21 (−99)	2208 (−62)	218 (−96)
*M. grande*	4453 [0.42]	0.53	10,023 (125)	8946 (101)	12,233 (175)	6001 (35)	4655 (5)	2768 (−38)	8695 (95)	7841 (76)	9606 (116)	4169 (−6)	8790 (97)	4089 (−8)
*M. hookeri*	8498 [0.33]	0.46	26,881 (216)	28,719 (238)	25,305 (198)	24,783 (192)	27,314 (221)	25,992 (206)	24,321 (186)	50,007 (488)	29,110 (243)	51,511 (506)	28,136 (231)	24,654 (190)
*M. orbiculare*	6451 [0.11]	0.33	0 (−100)	0 (−100)	0 (−100)	0 (−100)	0 (−100)	0 (−100)	0 (−100)	0 (−100)	0 (−100)	0 (−100)	0 (−100)	0 (−100)
*M. ovoideum*	3063 [0.37]	0.28	0 (−100)	0 (−100)	0 (−100)	0 (−100)	0 (−100)	0 (−100)	0 (−100)	0 (−100)	0 (−100)	0 (−100)	0 (−100)	0 (−100)
*M. parvifolium*	4027 [0.38]	0.21	0 (−100)	0 (−100)	0 (−100)	0 (−100)	0 (−100)	0 (−100)	0 (−100)	0 (−100)	0 (−100)	0 (−100)	0 (−100)	0 (−100)
*M. petiolatum*	18,259 [0.47]	0.82	23,143 (27)	22,070 (21)	21,768 (19)	22,594 (24)	23,702 (30)	21,813 (19)	18,582 (2)	21,208 (16)	18,645 (2)	20,258 (11)	22,870 (25)	26,277 (44)
*M. procerum*	3146 [0.78]	0.43	0 (−100)	0 (−100)	0 (−100)	0 (−100)	0 (−100)	0 (−100)	0 (−100)	0 (−100)	0 (−100)	0 (−100)	0 (−100)	0 (−100)
*M. rhinophyllum*	14,152 [0.11]	0.50	26,881 (90)	28,719 (103)	25,305 (79)	24,783 (75)	27,314 (93)	25,992 (84)	24,321 (72)	12,861 (−9)	29,110 (106)	24,654 (74)	28,136 (99)	25,561 (81)
*M. rivulare*	5306 [0.85]	0.43	5320 (0.2)	11,032 (107)	0 (−100)	5382 (1)	15,208 (186)	12,510 (135)	9091 (71)	11,851 (123)	11,363 (114)	6003 (13)	19,537 (268)	11,881 (123)
*M. rostratum*	7753 [0.60]	0.53	2854 (−63)	11,851 (52)	4511 (−41)	4020 (−48)	15,208 (96)	12,510 (61)	3598 (−53)	3598 (−53)	5312 (−31)	6379 (−17)	6379 (−17)	5170 (−33)
*M. rotundatum*	4260 [0.59]	0.37	0 (−100)	0 (−100)	0 (−100)	0 (−100)	0 (−100)	0 (−100)	0 (−100)	0 (−100)	0 (−100)	0 (−100)	0 (−100)	0 (−100)
*M. royenii*	15,993 [0.40]	0.62	35,229 (120)	37,671 (136)	34,318 (115)	43,038 (169)	36,760 (130)	42,842 (168)	33,737 (111)	42,296 (164)	38,346 (140)	34,709 (117)	36,644 (129)	43,509 (172)
*M. sylvaticum*	17,843 [0.48]	0.71	34,416 (93)	23,225 (30)	32,147 (80)	24,119 (35)	24,603 (38)	30,808 (73)	15,454 (−13)	32,032 (80)	26,742 (50)	27,008 (51)	29,088 (63)	16,963 (−5)
*M. umbellatum*	19,077 [0.48]	0.81	18,068 (−5)	19,841 (4)	16,321 (−14)	20,420 (7)	17,985 (−6)	16,767 (−12)	19,749 (4)	17,320 (−9)	18,850 (−1)	19,743 (3)	19,142 (0)	16,637 (−13)
*M. urceolatum*	9640 [0.64]	0.53	0 (−100)	0 (−100)	0 (−100)	0 (−100)	0 (−100)	0 (−100)	0 (−100)	0 (−100)	0 (−100)	0 (−100)	0 (−100)	0 (−100)
*M. varians*	7314 [0.36]	0.49	7853 (7)	14,282 (95)	15,808 (116)	25,599 (250)	15,993 (118)	9294 (27)	16,189 (121)	18,431 (150)	15,296 (109)	47,703 (552)	22,625 (209)	18,039 (146)

Note

BCC‐CSM1‐1 and MIROC5 are abbreviated as B and M, respectively.

Niche breadth values used to determine endemic categories are also provided. Cohen's KAPPA statistics which measure model performance are provided in square brackets in the current suitable habitats column and % changes (+ values are suitable habitat increases and – values are habitat reductions) are provided in parentheses in Future suitable habitats columns.

### 3.3 Future projections

**FIGURE 3:**
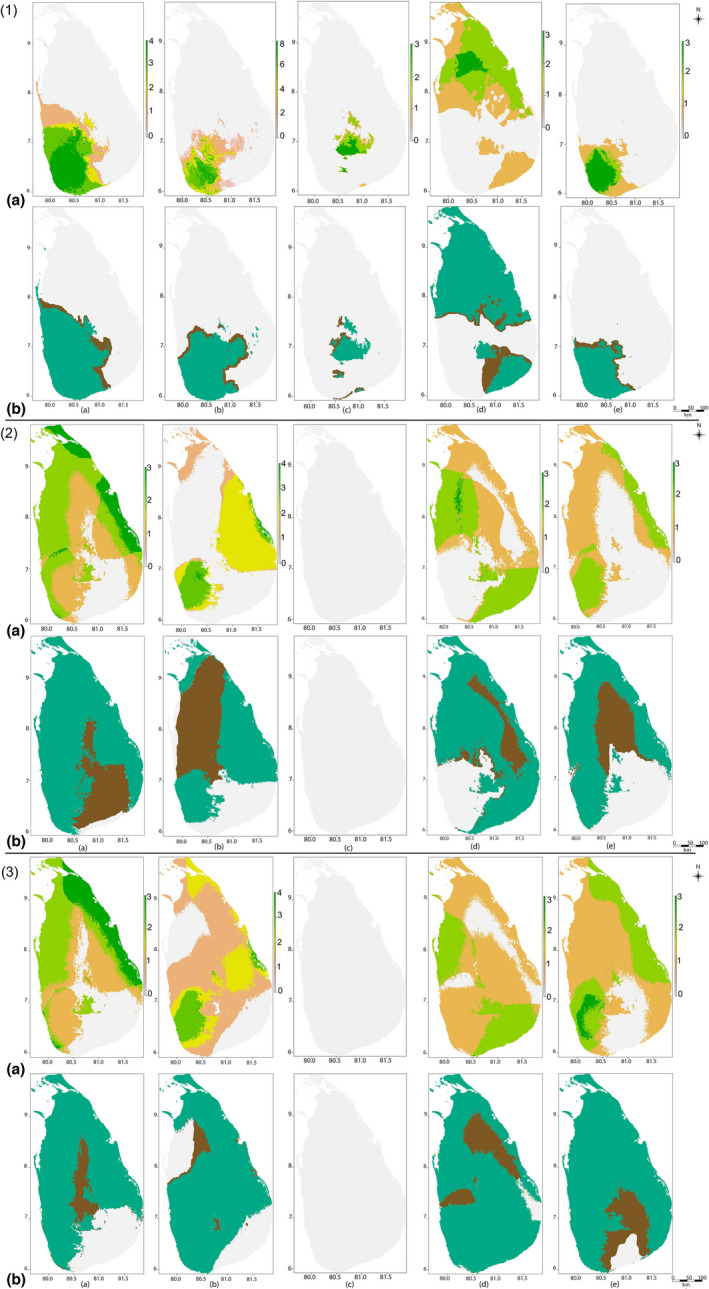
Overlapping suitable habitats of endemic *Memecylon* categories under current climate and future climate scenarios. Three major panels show: top‐panel/(1) Overlapped current suitable habitats, mid‐panel/(2) Overlapped suitable habitats in 2050 (MIROC5‐RCP2.6 scenario), and bottom‐panel/(3) Overlapped suitable habitats in 2070 (MIROC5‐RCP2.6 scenario). In each of the three panels, endemic categories are provided as (a) wide endemics; (b) narrow endemics restricted to lowland; (c) narrow endemics montane zone; (d) nonendemic *Memecylon* in the dry zone; and (e) nonendemics in the wet zone. In each of the three panels, (a) shows the overlapping suitable habitats and (b) shows the uncertainty maps of each endemic category. The color spectrum at the right of the maps in the map set (a) shows the number of species in each grid cell. Color code in the maps in the map set (b) shows the uncertainty of the prediction of species presence: turquoise areas that always predict species is present, brown areas with different predictions of species presence or absence, gray areas that species is absent

Responses of *Memecylon* to different future climate scenarios are variable (Table 4). MESS analysis resulted in negative changes, indicating that future climate scenarios result in significantly altered conditions at present‐day points of occurrences (Dryad). Overall, eight species were predicted as consistently losing habitat, while six species gained habitat under all future scenarios. All species in the narrow endemic montane zone category, three species in the narrow endemic lowland category (*Memecylon orbiculare* Thwaites, *Memecylon procerum* Thwaites, and *Memecylon rotundatum* Thwaites (Cogniaux)), and one wide endemic wet zone species (*Memecylon urceolatum* Cogniaux) were predicted to undergo a total decrease under future climate scenarios (Table 4). The predictor variable, precipitation of coldest quarter (bio19), was an important determinant of suitable habitat for all *Memecylon* species that completely lost all suitable habitats under future climate scenarios. *Memecylon hookeri* Thwaites had the largest increases in suitable habitat under climate change models (Table 4). The climate model MIROC5 showed more changes (habitat gains, shifts, and losses) than BCC‐CSM1‐1 (Table 4). Interestingly, many areas currently unsuitable are predicted to become increasingly suitable for *Memecylon*, while some currently suitable regions will become unsuitable in the future (Figure 3 and Table 4). For instance, lowland narrow endemic, wet zone nonendemic, and wide endemic categories showed potential eastward habitat shifts where the species belonging to these categories are currently absent. The habitat changes explained above will occur as early as 2050. As expected, most species showed a greater percentage of habitat change in 2070 compared to 2050 (Table 4).

### 3.4 Patterns in endemic categories

In all endemic categories, richness areas show habitats where all species overlap, based on the total number of species within a grid cell (e.g., richness area maps in Figure 3, wide endemics show areas where from 0–4 species overlap, narrow endemic lowland shows areas from where 0–8 species overlap, etc.). In the current Sri Lankan forests, richness maps of suitable habitats of *Memecylon* mainly occur in the lowland wet zone (Figure 3, top panel). In the lowland wet zone, we found that 15 of the 21 *Memecylon* species were predicted to have suitable habitats, and all species can be found together in some areas of suitable habitats in this zone (Figure 3, top panel). We identified that suitable habitats were absent for all categories of *Memecylon* in the southeast dry zone (Figure 3, top panel).

Examining how climate change might impact richness maps of the various endemic categories, we found that for montane narrow endemics, there was a complete loss of suitable habitat even under the optimistic MIROC5 (RCP 2.6) model shown in Figure 3. For wide endemics, we found that there were reduced suitable habitats that captured areas of high species richness, especially in 2050 models. For nonendemic categories, new areas that capture multiple species within those categories emerged, suggesting that environmental conditions improve for these species, assuming that they can disperse and track changes over time.

### 3.5 Gap analysis

**FIGURE 4:**
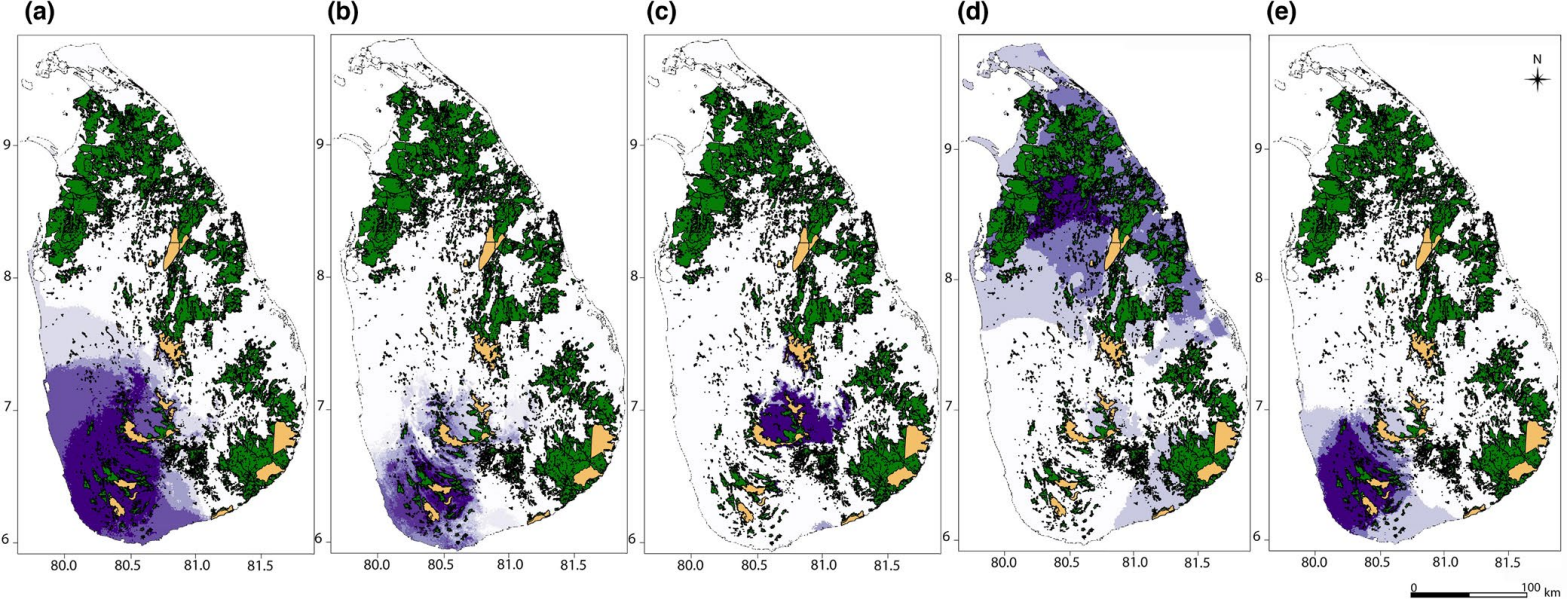
Over‐laid forest cover (source: Rathnayake et al., 2020) and protected areas maps (source: WDPA) with the current richness areas (areas show habitats where all species overlap in darkest purple) of (a) wide endemics; (b) narrow endemics restricted to lowland; (c) narrow endemics montane zone; (d) nonendemic *Memecylon* in dry zone; and (e) nonendemics in the wet zone. Suitable habitats of individual species are shown in purple shades. The darkest purple represents the highest overlap areas for species richness; green represents forest cover; and orange represents protected areas

**FIGURE 5:**
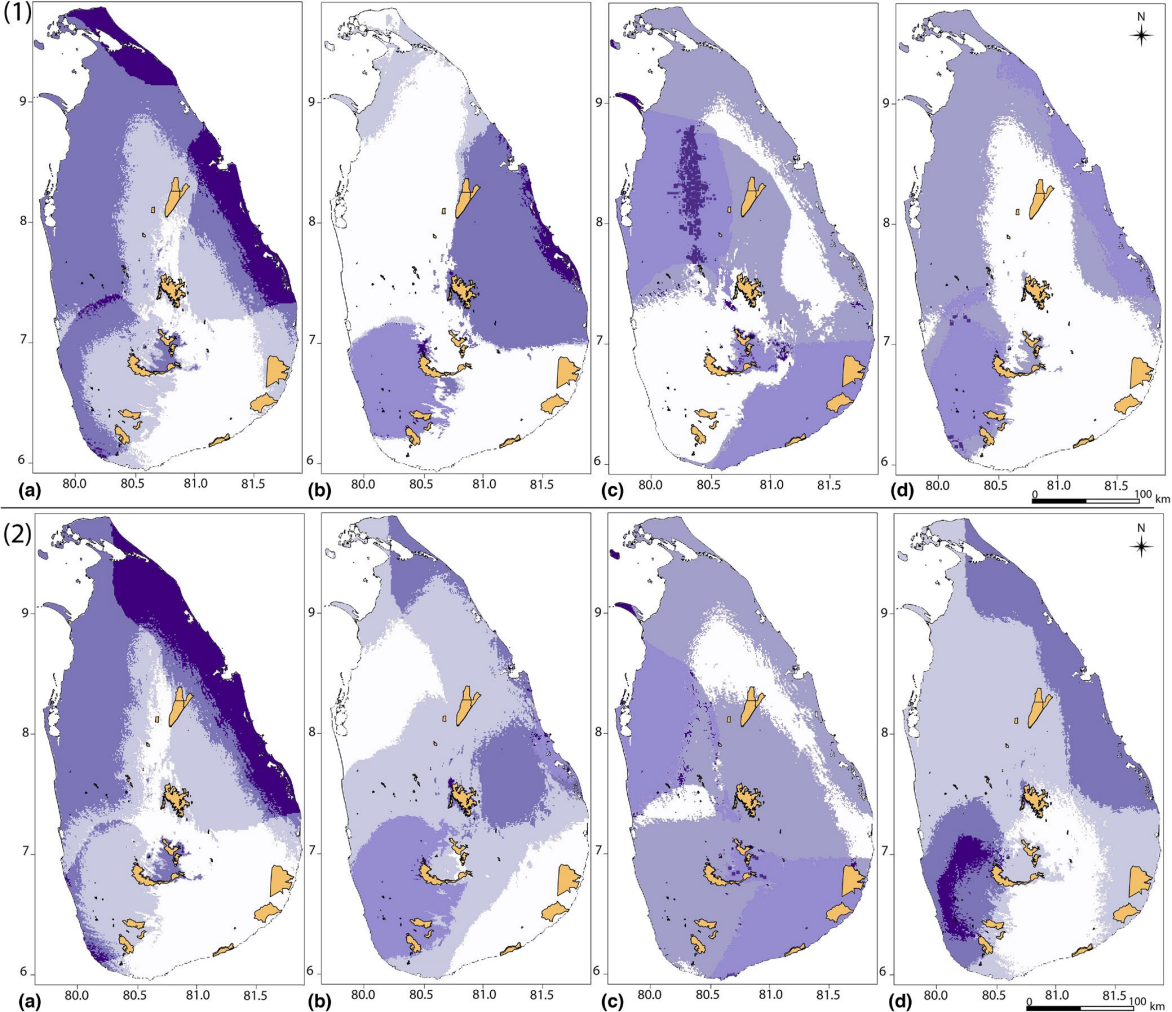
Over‐laid protected areas maps (source: WDPA) with the future richness areas (areas show habitats where all species overlap in darkest purple) of (a) wide endemics; (b) narrow endemics restricted to lowland; (c) nonendemic *Memecylon* in the dry zone; and (d) nonendemics in the wet zone; narrow endemics in the montane zone are not shown as no suitable habitat is predicted to occur in future climates. The top panel shows the suitable habitats in 2050 (MIROC52.6). The bottom panel shows the suitable habitats in 2070 (MIROC52.6). Suitable habitats of individual species are shown in purple shades. The darkest purple represents the highest overlap areas for species richness, and orange represents protected areas

The overlay of the richness area map (the area that captures the maximum number of species within each category as shown in darkest green in Figure 3) for each category of *Memecylon* with the protected area and forest maps resulted in information relevant for conservation. Land area extracted from protected areas and land‐cover maps are provided in Dryad. The area estimations after superimposing these extracted areas with *Memecylon* richness areas are provided in Figures 4 and 5 and Table 5. Using land‐cover maps, the current forest was found to total 17,723 km^2^ (~27% of total land cover in forest vegetation). When the species richness areas (darkest purple in Figure 4) for each category of *Memecylon* under current climate conditions were superimposed with forest cover, 17.8% of the richness area of wide endemic *Memecylon* overlapped with forest cover, while richness area of lowland narrow endemic *Memecylon* coincided with 43% of forest cover. The richness area of the montane narrow endemic *Memecylon* category overlapped with 46.3% of the richness area of forest cover and that of the nonendemic dry zone *Memecylon* category overlapped with 19.9% of the area of forest cover and that percentage was 19.8% for nonendemic wet zone *Memecylon*. The estimated area of the total protected land was about 2026 km^2^ and land allocated for conservation forests was 1209 km^2^. When the protected area map containing all conserved forests was superimposed with the richness areas of each category at the current distribution (Figure 4), the overlapping area of the montane narrow endemic category was the best protected (26.5%).

**TABLE 5:** Gap analysis using protected area map, land‐use map, and richness area (represent only those areas with a maximum number of overlapping species) maps

Current					Future					
					(2050‐MIROC5‐2.6)			(2070‐MIROC5‐2.6)		
Category	Richness area (km^2^)	Extent within all protected (km^2^)	Extent within conservation forest (km^2^)	Extent within forests (km^2^)	Richness area (km^2^)	Extent within all protected (km^2^)	Extent within conserved forests (km^2^)	Richness area (km^2^)	Extent within all protected (km^2^)	Extent within conserved forests (km^2^)
Wide endemic	7235	425	108	1288	6875	7	7	9463	22	22
Lowland narrow endemic	291	35	13	125	356	0.7	0.06	99	0.2	0.09
Montane narrow endemic	1326	352	180	615	N/A	N/A	N/A	N/A	N/A	N/A
Nonendemic dry zone	3946	106	93	786	1070	10	8	46	0.9	0.9
Nonendemic wet zone	4239	250	47	840	19	0	0	2059	16	6

Note

Richness areas = Richness area of the category. Extent within all protected = Extent of each category overlapped with all types of protected lands in the protected area map. Extent within conservation forest = Extent of each category overlapped with conservation forests in the protected area map. Extent within forests = Extent of each category within Forest in the land‐cover map. N/A = not applicable due to total loss of overlapping areas.

Estimates are provided as area overlapping lands in each category and the extent of each category within protected lands and forests. Both protected area map and land‐use map are used for current models, but only protected area map is used for future models.

When the same superimposition was performed with the richness areas of each category in the future (2050 and 2070), the percentage overlap with all types of protected lands decreased (Figure 5 and Table 5). Indeed, none of the “richness areas” for the nonendemic wet zone category overlap with protected lands in 2050. As there is a total loss in narrow endemics restricted to the montane zone in future projections, this category was not included in the gap analysis.

**FIGURE 6:**
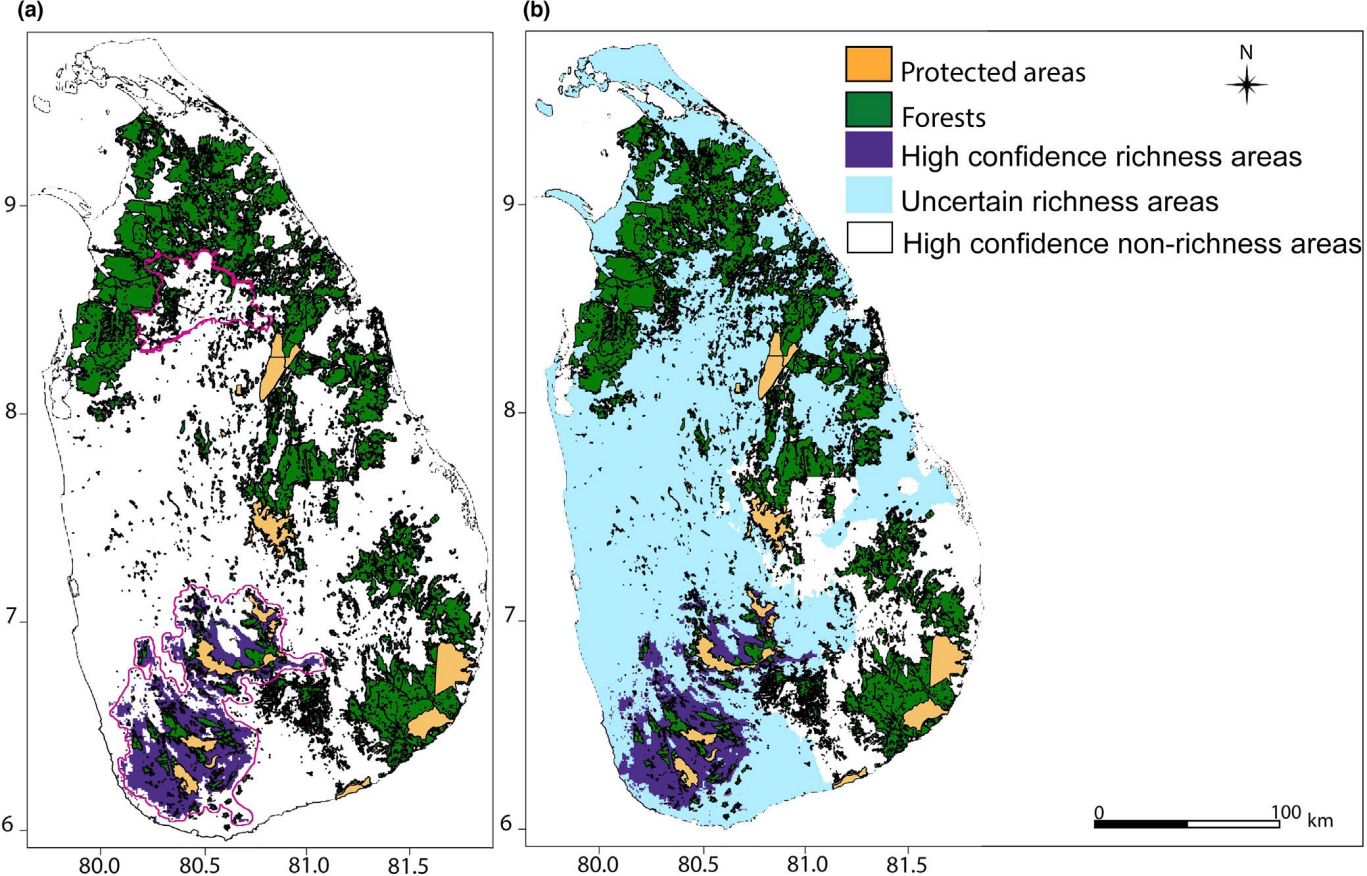
Areas recommended for conservation. (a) Conservation recommendation map: current richness areas (areas show habitats where the highest number of species overlap) of endemism categories, forest cover (source: Rathnayake et al., 2020), and protected areas maps (source: WDPA) are superimposed. The forests which require conservation are within the areas demarcated in magenta. These areas include both the purple areas within these demarcations represent high confidence richness areas (these are obtained from multiple model iterations and purple shows all model iterations for the highest number of species predict present) and richness area of dry‐zone nonendemic *Memecylon* in the northern part of the island which is not within the high confidence richness areas. (b) Uncertainty map: The uncertain richness areas (at least one model iteration for all species predicts presence) are shown in blue and the high confidence nonrichness areas (at least one species is predicted to be absent in all model iterations) are shown in white

There are several opportunities to improve conservation outcomes for *Memecylon* (Figure 6). High confidence richness areas of *Memecylon* which are found in the southwest and central regions of Sri Lanka are protected to some extent, but require much attention because while currently suitable habitats of endemic *Memecylon* are concentrated in these areas, significant areas of forest remain unprotected. Nonendemic dry zone *Memecylon* showed a richness area in the north‐central and eastern regions of the island (Figures 3 and 4). These species were within the uncertain richness areas (Figure 6). However, forests in this area are the least protected under the current conservation policies. We recommend conservation of these forests because *Memecylon* that exist only in the dry zone showed a richness area restricted to this part of the island.

## 4 DISCUSSION

Distribution data of *Memecylon* used for the analysis covered the entire current range within Sri Lanka and represent the variability of climatic conditions in which *Memecylon* occurs on this island. Niche models of other organisms of Sri Lanka have provided valuable insights into the patterns of current potential distribution (Amphibians—Wijayathilaka et al., 2018; primates—Hettiarachchi et al., 2018; Cycads—Mudannayake et al., 2019); however, future predictions of the organisms in the island are, thus far, totally unavailable. Therefore, our study is the first complete prediction of future range shifts of a woody angiosperm taxonomic group, *Memecylon*, in Sri Lanka, contributing information about responses of an island‐dwelling group to climate fluctuation events.

### 4.1 Response to climate change

The overall current distribution showed that about 60,016 km^2^ of the total area in Sri Lanka is potentially suitable for *Memecylon*. These suitable habitats cover all climate and elevation zones in Sri Lanka except the southeast dry zone (Figure 3, top panel). The current models revealed that most of the known *Memecylon* occurrence points are within suitable habitats, while there are still some areas potentially suitable for colonization.

Our results demonstrate that future climate change scenarios will lead to varied responses of the amount of suitable habitats for *Memecylon*. The total loss of suitable habitats of *Memecylon* in montane regions under future climate scenarios indicates that *Memecylon* species occupying mountains will be especially affected by climate change. The unique climatic conditions in the mountains of Sri Lanka (Jayalal et al., 2017; Ruklani & Rubasinghe, 2021; Werner, 1995) provide limited opportunities for growth and survival. Our results also suggested that under future predicted climate change, most *Memecylon* with small fundamental niches (narrow endemics) are susceptible to habitat loss (Figure 3 and Dryad). Our results supported the hypothesis that endemic categories would be highly affected by climate change and montane endemics were the most susceptible group. Habitat gain with climate change was observed for *Memecylon* distributed in all three zones and at low to medium elevations. The original shapefiles for the species gaining habitat were the largest of any species used in this analysis, and they overlapped considerably with one another. Therefore, one possible explanation for why these species show an increase or are little affected in suitable habitats under projected future climate change is likely due to their widespread distribution and broad ecological niche tolerances.

Large changes are already evident by 2050 for both suitable habitat areas and species richness areas of *Memecylon*. This prediction is congruent with recent studies of the other organisms in different regions of the world (Ahmad et al., 2019; Puchałka et al., 2021). However, with limited knowledge about the dispersal ability of *Memecylon*, it is not possible to predict how they will be able to migrate to new areas in the near future. This information is critical to understand the need to consider using human‐assisted dispersal mechanisms (Hoegh‐Guldberg et al., 2008) into potentially suitable areas as conservation intervention strategies. A more detailed study on *Memecylon* taking into account dispersal biology, genetic diversity, and phenology of these species would help to elucidate these patterns (CaraDonna & Inouye, 2015; Cotto et al., 2017; Kearney & Porter, 2009), but our investigation into climatic and abiotic drivers provides initial insights regarding response to a changing climate.

### 4.2 Conservation prioritization

Another objective of this study was to identify gaps in conservation among *Memecylon* endemic categories within the current protected area network. Our results suggest that montane narrow endemic and lowland narrow endemic categories of *Memecylon* are quite well represented in the protected area network with 26.5% and 12.1% of the land protected, respectively; these values respectively exceed and closer to the global land protection average of 12.7% (UNEP‐WCMC & IUCN, 2020). Additionally, under Aichi target 11, a goal of 17% of land under protection level is required, including areas of particular importance for biodiversity (Convention on Biological Diversity, 2010). Although 30% terrestrial protected area coverage is recorded from Sri Lanka (UNEP‐WCMC, 2017), this effort should spread to capture the diversity within Sri Lanka, specifically habitats similar to *Memecylon* richness areas.

Under future climates, a significant reduction of richness areas within the current protected areas was observed for all endemic categories (Figures 4 and 5). Here, only the current suitability is considered for conservation recommendations because planning for future climate scenarios is problematic given uncertainty regarding models and policies that might mitigate (or not) climate change impacts. As the future predictions indicate habitat change of *Memecylon* in 2050, there is an urgent need to implement conservation management for vulnerable *Memecylon* categories. In particular, rare and endemic species of Sri Lankan *Memecylon* warrant conservation attention due to the predicted habitat loss inferred from this study. To address and mitigate these losses, various other conservation parameters, such as estimating the land cost, regional versus global conservation priorities, and conservation risks, should also be taken into account (Butt et al., 2020; Naidoo & Ricketts, 2006). Moreover, as sample sizes were generally low for many species studied here, conservation planning and actions require further detailed spatial analyses to identify both problems and opportunities in a regional and local socio‐ecological context.

### 4.3 Future directions for studies of *Memecylon* ecological niches

We used only a subset of total Sri Lankan *Memecylon* as we eliminated species with few occurrence data and species found from a single national park. In addition, we identified a significant undiscovered diversity of Sri Lankan *Memecylon* during fieldwork. In future studies, this diversity should be taken into account. Also, low sample sizes and potential bias in sampling associated with inadequately capturing the environmental conditions in which the species occurs call for additional fieldwork and further spatial analyses. Further, we used only 12 future climate models among all possible future scenarios (Hijmans et al., 2005). Moreover, examining these niche differences in the context of phylogenetic relationships may help us understand the factors that have led to the diversification of *Memecylon* within the island and to infer ancestral niches. However, Sri Lankan *Memecylon* is not monophyletic; instead, it includes *Memecylon* from India, Andaman, and the Seychelles (Amarasinghe, Joshi, et al., 2021). To understand the ancestral niches of Sri Lankan *Memecylon*, niche models should be generated from all these geographical regions in South Asia where *Memecylon* occurs and analyzed in a phylogenetic framework. However, incomplete information on occurrence data and identification errors of a large number of *Memecylon* specimens from the other South Asian regions impeded constructing niche models and understanding ancestral niches. Sri Lanka has a rich diversity of soils distributed across the island (Wimalasiri et al., 2020); however, information about the soil requirements of *Memecylon* is totally lacking. Therefore, future studies should also include soil data to understand the abiotic niches of *Memecylon*.

Our findings will help clarify general patterns of woody plants occupying habitats in Sri Lanka and provide data to inform conservation strategies on this island. Given the expected significant changes in future suitable habitats of this plant group, the reduction of the area occupied by the species in the richness areas will be intensified unless species are able to adapt to the future climate change or conservation measures are implemented.

## CONFLICT OF INTEREST

All authors declare no conflict of interest.

## AUTHOR CONTRIBUTIONS

**Prabha Amarasinghe:** Conceptualization (equal); Data curation (lead); Formal analysis (equal); Funding acquisition (equal); Investigation (lead); Methodology (lead); Resources (equal); Writing – original draft (lead); Writing – review & editing (equal). **Narayani Barve:** Conceptualization (equal); Formal analysis (equal); Methodology (supporting); Writing – review & editing (equal). **Hashendra Kathriarachchi:** Resources (equal); Writing – review & editing (equal). **Bette Loiselle:** Conceptualization (equal); Methodology (supporting); Supervision (equal); Writing – review & editing (equal). **Nico Cellinese:** Conceptualization (equal); Funding acquisition (equal); Project administration (lead); Resources (equal); Supervision (equal); Writing – review & editing (equal).

## Data Availability

Species occurrence data, niche models (MaxEnt output and results of MESS analysis), and scripts are available from Dryad (https://doi.org/10.5061/dryad.sxksn034f) (Amarasinghe, Barev, et al., 2021).
